# Tapping the rhizosphere metabolites for the prebiotic control of soil-borne bacterial wilt disease

**DOI:** 10.1038/s41467-023-40184-2

**Published:** 2023-07-26

**Authors:** Tao Wen, Penghao Xie, Hongwei Liu, Ting Liu, Mengli Zhao, Shengdie Yang, Guoqing Niu, Lauren Hale, Brajesh K. Singh, George A. Kowalchuk, Qirong Shen, Jun Yuan

**Affiliations:** 1 Jiangsu Provincial Key Lab for Organic Solid Waste Utilization, Jiangsu Collaborative Innovation Center for Solid Organic Wastes, Educational Ministry Engineering Center of Resource-saving fertilizers, Nanjing Agricultural University, Nanjing, 210095 China; 2 Key Laboratory of Green Intelligent Fertilizer Innovation, MARD, Sinong Bio-organic Fertilizer Institute, Nanjing, 210000 China; 3 Hawkesbury Institute for the Environment, Western Sydney University, Penrith, NSW 2753 Australia; 4 USDA, Agricultural Research Service, San Joaquin Valley Agricultural Sciences Center, Parlier, CA USA; 5 Ecology and Biodiversity Group, Department of Biology, Institute of Environmental Biology, Utrecht University, Padualaan 8, 3584 CH Utrecht, The Netherlands

**Keywords:** Microbiome, Secondary metabolism

## Abstract

Prebiotics are compounds that selectively stimulate the growth and activity of beneficial microorganisms. The use of prebiotics is a well-established strategy for managing human gut health. This concept can also be extended to plants where plant rhizosphere microbiomes can improve the nutrient acquisition and disease resistance. However, we lack effective strategies for choosing metabolites to elicit the desired impacts on plant health. In this study, we target the rhizosphere of tomato (*Solanum lycopersicum*) suffering from wilt disease (caused by *Ralstonia solanacearum*) as source for potential prebiotic metabolites. We identify metabolites (ribose, lactic acid, xylose, mannose, maltose, gluconolactone, and ribitol) exclusively used by soil commensal bacteria (not positively correlated with *R*. *solanacearum*) but not efficiently used by the pathogen in vitro. Metabolites application in the soil with 1 µmol g^−1^ soil effectively protects tomato and other *Solanaceae* crops, pepper (*Capsicum annuum*) and eggplant (*Solanum melongena*), from pathogen invasion. After adding prebiotics, the rhizosphere soil microbiome exhibits enrichment of pathways related to carbon metabolism and autotoxin degradation, which were driven by commensal microbes. Collectively, we propose a novel pathway for mining metabolites from the rhizosphere soil and their use as prebiotics to help control soil-borne bacterial wilt diseases.

## Abstract (web summary)

Prebiotics can be used to encourage beneficial organisms. Here, the authors select rhizosphere metabolites that can be used as prebiotics to reduce the effect of the plant pathogen *Ralstonia*.

## Introduction

Soil-borne plant diseases cause devastating losses of plant yield and farm profitability across the globe^1–3^. Conventional chemical strategies to combat such diseases have proven to be ineffective, unsustainable and come at a severe environmental cost. Thus, biocontrol alternatives have been proposed as a more environmentally friendly alternative to chemical pesticides in agricultural ecosystems^4^. Microbial inoculants, however, are often unstable or lose efficacy over time due to poor adaptation to the environment where their activities are required. In agricultural soils, inoculated microbial agents are subjected to multiple environmental stresses and can be seen as “invaders” that must compete for niches with populations within the native microbiome, potentially reducing their effectiveness^5^. Soil microbial communities naturally possess a range of organisms that can antagonize or outcompete pathogens, thereby constraining the outbreak of diseases^5,6^. It has therefore been suggested that effective biocontrol might be achieved via in situ manipulation of the resident rhizosphere microbiome to enhance the abundance and activities of beneficial microbes^7,8^. To date, most prebiotics has been polysaccharides that bring host benefits by promoting the abundance or activity of beneficial microbes in a given system^9–11^. While prebiotics targeting human/animal gut microbiomes have been widely explored^12^, efforts designed for prebiotics to enhance plant microbiomes has received rather little attention. As described in some studies, plant growth-promoting rhizobacteria (PGPR) can serve as the plant equivalent of gut probiotics, while certain substrates or additives that modify the composition/diversity of plant microbiomes can function similarly to prebiotics in humans^13^. This opens up a realm of possibilities for the utilization of prebiotics in agriculture practices. For instance, organic amendments, such as composts derived from plant and animal residues, contain a diverse range of complex carbohydrates that act as prebiotics, nurturing beneficial microorganisms in soils^14^. Additionally, some beneficial root exudate components can also function as prebiotics, enhancing intercommunications between plants and beneficial microorganisms^15^.

The soil zone on and around plant roots, collectively called the rhizosphere, is a critical interface for plant nutrient acquisition, stress tolerance, and disease suppression. As a source of carbon and nitrogen, rhizosphere metabolites can recruit beneficial microbes to antagonize phytopathogens^16^. For example, malic acid has been linked with the recruitment of *Bacillus subtilis* in *Arabidopsis thaliana*^17^, and our previous study indicated that fatty acids and amino acids could promote the abundance of beneficial *Pseudomonas* strains in soil^18^. The beneficial impacts of such recruited microbes may stem from direct metabolic antagonism (e.g., via antibiotics), competition for resources^19^, indirect effects via suppression of populations that facilitate pathogen growth^20^, or via induction of plant defenses. Most importantly, the composition of metabolites in the rhizosphere has been shown to change when confronted by plant pathogens, and such shifts linked to development of disease-suppressive soils via the recruitment of beneficial microbial populations^9,10^. Therefore, we propose that metabolites that preferentially existed in the rhizosphere in response to pathogen attack may serve as a natural library for potentially valuable prebiotics. Harnessing such natural prebiotic compounds could help develop more effective strategies for targeted rhizosphere microbiome engineering to better manage soil-borne diseases better. When applying prebiotic metabolites, it is also important to consider potential disturbance of the resident microbial community, as highly disturbed communities may be susceptible to invasion by another pathogens. Indeed, the application of certain compounds has been shown to decrease microbial community diversity or disturb the soil microbiome^18^. Studies have shown that increasing the variety of applied compounds alleviated declines in soil microbial diversity^21,22^. Thus, the application of multiple prebiotic compounds might represent a more suitable strategy than the use of any single prebiotic compound.

In this study, we sought to develop a systematic strategy for the development of prebiotics by focusing on natural rhizosphere metabolites. We conducted a comparative analysis of the metabolic profiles of rhizosphere samples from diseased versus healthy plants grown in a soil conductive to bacterial wilt disease. We argued that the organic metabolites that were more abundant in the healthy plant rhizosphere might be linked with the recruitment of beneficial microbes, thereby contributing to the observed plant health status.

Afterward, we delved into the mechanisms behind the stimulation of commensal microbes for disease suppression using both in vitro (e.g., carbon utilization test) and in situ tests (synthetic microbial consortia approach). To validate our findings in tomato, we also examined the effects of the prebiotics on other plant species. Finally, our metagenomic analysis allowed us to gain a deeper understanding of the mechanisms underlying prebiotic-driven rhizosphere microbial ecology, and how it can be used to enhance disease resistance in plants. Overall, we report a novel strategy for developing rhizosphere prebiotics that can bring directional change in rhizosphere microbiomes to address biotic stresses of plants.

## Results

### Metabolomic analysis of the rhizosphere soil of healthy and diseased tomato plants

**Fig. 1:**
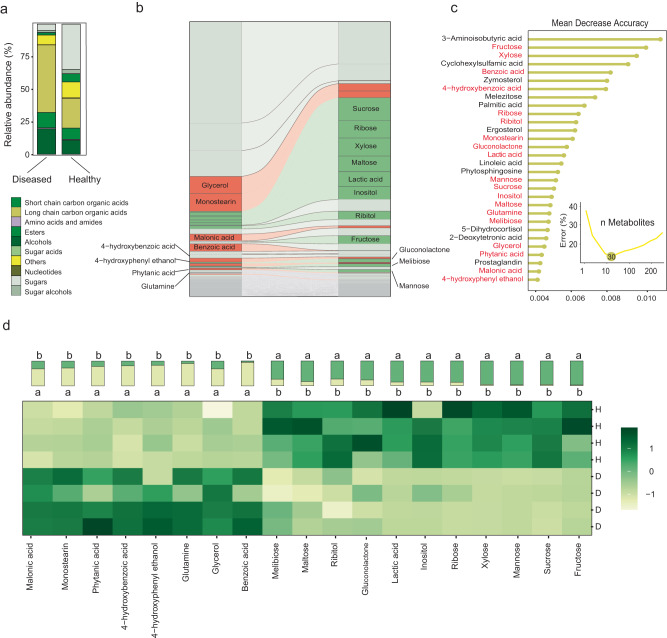
Metabolite profiles of rhizosphere soil in diseased and healthy plants. **a** Rhizosphere metabolites profiles. Metabolites were grouped according to their chemical properties and plotted as stacked column charts; **b** The flow plot showed the relative abundances of individual metabolites in diseased and healthy samples. Each line represented one metabolite, and the vertical axis indicates the relative abundance of all identified 216 rhizosphere metabolites. The most abundant metabolite plotted at the top of the diagram. Metabolites significantly enriched in healthy samples by GLM analysis were highlighted in green. Significantly enriched metabolites in diseased samples are marked in red, and others were gray; **c** The top 30 marker metabolites were identified by applying random forest classification. The marker metabolites are ranked in descending order of importance concerning the model’s accuracy. The inset represents ten-fold cross-validation error as a function of the number of input metabolites used to differentiate diseased and healthy in order of variable importance; **d** Heatmap revealed differential abundance of metabolites in healthy and diseased Samples. A total of 19 metabolites were counted, with 11 rhizosphere metabolites abundances were higher in the rhizosphere of healthy tomato plants, and eight rhizosphere metabolites abundances were higher in the rhizosphere of diseased tomato plants.

In our field experiment, tomato plants were cropped in a bacterial wilt disease conductive soil, where both healthy and bacterial wilt disease infected plants at the fruiting stage were collected for the investigation of their rhizosphere metabolites. A total of 216 compounds were identified in the rhizosphere soil metabolomes across all samples as informed by the analysis of gas chromatography-time of flight mass spectrometry (GC-TOF-MS). Principal components analysis revealed that the metabolite composition in samples was distinct in the healthy versus diseased plants (R = 0.785, *p* = 0.026, Adonis), as also indicated by PCA ordination with the X and Y axe explaining 44.68% and 16.34% of the total variation, respectively (Supplementary Fig. 1). Among the 216 compounds, 32 sugars, six sugar alcohols, five sugar acids, 22 short chain carbon organic acids, 23 long chain carbon organic acids, 14 esters, 23 alcohols, 18 amino acids and amides, two nucleotides, and 71 others were included. The results demonstrated a large diversity of metabolites in the rhizosphere and their potential to interact with the plethora of soil microbes. Importantly, sugars were more abundant in the healthy tomato rhizosphere (34.8%) than in the diseased plant rhizosphere (4.75%); while in contrast, long chain organic acids were detected in larger abundance in the diseased plant rhizosphere (51.9%) compared to that of the healthy plants (21. 9%) (Fig. 1a and Supplementary Table 1).

We performed generalized linear model (GLM) analyses for each metabolite, and 79 metabolites significantly (*p* < 0.05, Turkey-HSD) differed in relative abundance between the diseased and healthy rhizosphere soils, among which 54 metabolites had higher abundance in the diseased plants. In comparison, 25 metabolites had higher abundance in the healthy plants (Fig. 1b). Subsequently, the random forest analysis was employed to further identify the important metabolites among the 79 differential ones, which could be potentially harnessed for the development of plant prebiotics. The cross-validation results indicated that the random forest model exhibited optimal stability and accuracy when selecting top 30 metabolites. Subsequently, we decided on a selection of 19 metabolites, favored due to their relative ease of acquisition in pure form for subsequent experiments (Fig. 1c). Based on the results of GLM analysis and random forest model, 19 metabolites (11 abundant in healthy samples and eight abundant in diseased samples) were then selected to validate our hypothesis that the healthy plant rhizosphere-associated metabolites assemble a microbiome that leads to an enhanced plant disease tolerance. The selected metabolites with significant enrichment in healthy samples including sucrose, fructose, mannose, xylose, ribose, inositol, lactic acid, gluconolactone, ribitol, melibiose and maltose, and those enriched in diseased samples including benzoic acid, malonic acid, 4-hydroxybenzoic acid, phytanic acid, 4-hydroxyphenyl ethanol, glycerol, glutamine, and monostearin (Fig. 1d).

### Effects of differential compounds on plant disease incidence and the rhizosphere microbiome

**Fig. 2:**
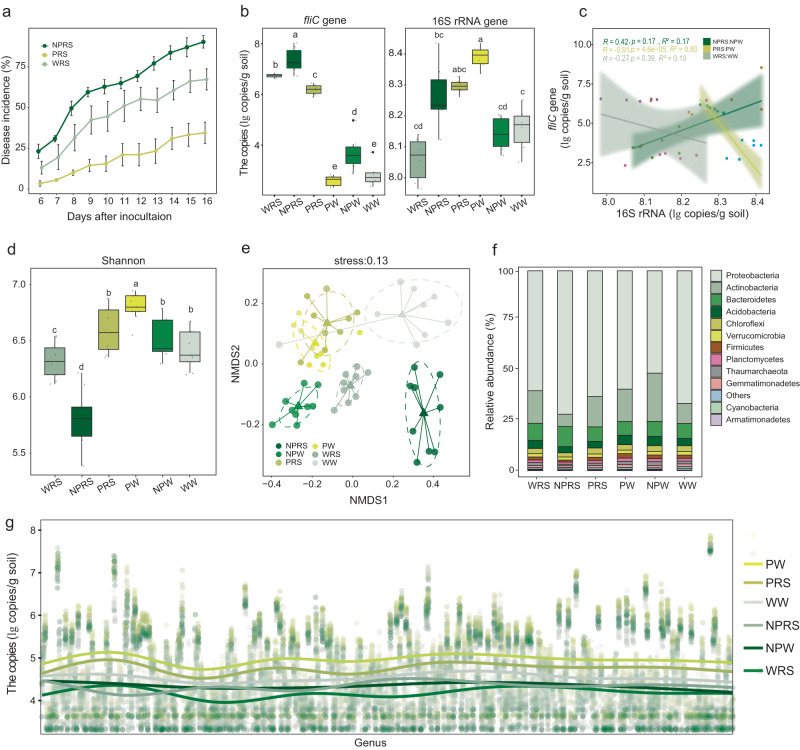
Effects of metabolites addition on plant disease occurrence and microbial community. **a** Disease incidence (bacterial wilt) in NRPS, PRS, and WRS treatments after pathogen inoculation was shown. All data are presented as mean ± standard error of the mean (SEM) in line graphs (*n* = 6 biologically independent samples); **b** Copy numbers of *fliC* and 16S rRNA genes among different rhizosphere samples. Horizontal bars within boxes represent the median. The tops and bottoms of boxes represent 75th and 25th quartiles, respectively. The upper and lower whiskers represent the range of non-outlier data values. Outliers were plotted as individual points. Different lowercase letters indicated significant differences among respective groups based on two-sided tests for multiple comparisons by Turkey HSD corrections (*t*-test, adjusted *p* < 0.05, *n* = 6 biologically independent samples); **c** Simple Linear regression analyses performed with the *fliC* and 16S rRNA genes. The solid lines represent regression lines and transparent areas represent 95% confidence interval. Two-sided *t*-test was used to test the significance of regression at 5% significance level; **d** *Alpha* diversity of the soil bacterial communities across the different treatments. Shannon calculated using the normalized ASV table. Horizontal bars within boxes represent the median. The tops and bottoms of boxes represent 75th and 25th quartiles, respectively. The upper and lower whiskers represent the range of non-outlier data values. Outliers were plotted as individual points. Different lowercase letters indicated significant differences among respective groups based on two-sided tests by Wilcoxon rank-sum test followed by Dunn’s multiple comparison test (adjusted *p* < 0.05, *n* = 9 biologically independent samples); **e** Nonmetric multidimensional scaling (NMDS) analysis based on Bray–Curtis dissimilarity on the taxonomic profile (at the ASV level) of the bacterial communities; **f** stack bar plot depicting the relative abundances (%) of the major phyla present in the bacterial communities; **g** The absolute abundance of 470 genera negatively correlated/ uncorrelated with *R. solanacearum* varied across samples. Absolute abundances were converted by multiplying the 16S rRNA gene qPCR quantification results by the relative abundance of microorganisms. The horizontal coordinates represent the 470 genera, and the vertical coordinates represent the absolute abundance. The dots represent the values of absolute microbial abundance in different samples, and the curves represent the results of fitting the changes in microbial abundance. WRS water with pathogen, PRS prebiotics with pathogen, NPRS non-prebiotics with pathogen, PW prebiotics with water, NPW non-prebiotics with water, WW only water.

We designed a prebiotic cocktail comprised of the 11 metabolites described above that showed the most robust enrichment in the rhizospheres of healthy tomato plants. To avoid the decreasing of microbial diversity from single metabolite application, we then tested the ability of this cocktail to reduce bacterial wilt disease in tomato plants. The cocktail was amended in soil (collected from Changshu city, China (31°35′36.19″N, 120°54′54.93″E), no tomato cultivation history) in four doses (once a week for four weeks) at 50 µmol per plant along with *R. solanacearum* (5 × 10^8^ CFU per plant) or sterile water in a greenhouse experiment. Eight significantly enriched metabolites (benzoic acid, malonic acid, 4-hydroxybenzoic acid, 4-hydroxyphenyl ethanol, glycerol, glutamine, monostearin and phytanic acid) in the diseased samples were added in the mix with the same dosage as positive controls. Their impacts on tomato disease incidence were monitored over a 16-day timeframe. Prebiotic application significantly lowered disease incidence (32.0%) in the metabolites-applied treatment (prebiotics with pathogen; PRS) more than in the water with pathogen (WRS), while non-prebiotic metabolite application (NPRS) increased the incidence by 22.4% compared to WRS (water with pathogen) (65.6%) (Fig. 2a and Supplementary Table 2). Quantitative PCR analyses of the abundances of the *fli*C gene (encoding the flagella subunit of *Ralstonia solanacearum*) revealed a lower abundance of the pathogen (−8.5%) in the metabolites-treated samples (PRS, prebiotics with pathogen) than that in the control (WRS, water with pathogen) post pathogen application (Fig. 2b and Supplementary Table 3). Compared to the control (WRS), the application of NPRS increased the abundance of pathogen by 8.3%. Furthermore, the total bacterial abundances, as determined by qPCR targeting the 16S rRNA gene, were significantly higher in the PRS (prebiotics with pathogen) and PW (prebiotics with water) than that of non-prebiotics treated samples WRS (water with pathogen) and the water control (WW, only water) (Fig. 2b and Supplementary Table 3). Furthermore, with the samples in PRS (prebiotics with pathogen) and PW (water with pathogen) treatment, univariate linear regression analysis revealed a significant negative correlation between the pathogen load and the total bacterial abundance in the rhizosphere (R = −0.91; *p* < 0.05; R^2^ = 0.8), suggesting that a larger bacterial population was associated with pathogen inhibition in the tomato plant (Fig. 2c). The abundance of pathogen was not significantly correlated with total bacterial abundance in the other treatments (Fig. 2c).

We then evaluated changes in the soil bacterial community diversity and composition in response to prebiotic cocktail amendment using high-throughput 16S rRNA gene tag sequencing. Our results of 16S rRNA amplicon sequencing of the plant and soil samples indicated that the pathogen (*Ralstonia solanacearum*) was present in soil of all treatments. It showed the lowest relative abundance in the WW (only water) and PW (prebiotics with water) treatments, while exhibited the highest abundance in the NPRS (non-prebiotics with pathogen) and WRS treatments (water with pathogen) (Supplementary Fig. 2). The cocktail application significantly increased the bacterial community species richness and *alpha* diversity, based on Shannon indices (*p* < 0.05, Dunn’s), both in the presence (PRS vs. WRS) and absence (PW vs. WW) of the pathogen (Fig. 2d). Furthermore, NMDS analysis (using Bray–Curtis distance) exhibited apparent differences in soil microbial community structure among treatments (stress = 0.13; PERMANOVA *p* = 0.001, R = 0.687) (Fig. 2e). Communities that had received the prebiotic cocktail (PRS and PW) were distinct from the control communities (WRS and WW) and non-prebiotic (NPRS and NPW) communities. Two-factor PERMANOVA revealed that the pathogen and prebiotics application (alone and together) explained about 41% of the total variance in microbial community structure. Individually, prebiotic application explained approximately 28% of the community variance, while pathogen treatment explained a further 7% (Supplementary Table 4 and Supplementary Table 5). At the phylum level, prebiotic amendment significantly increased the relative abundance of Actinobacteria but reduced the abundance of Proteobacteria (Fig. 2f).

To investigate the impact of metabolites on the potential microbial correlations within the soil microbial community, we conducted co-occurrence microbial network analyses. Our findings demonstrate that the rhizosphere soil microbiome exhibits a high degree of stability (WW, only water) (Supplementary Fig. 3 and Supplementary Table 6). The network stability decreases upon the addition of non-prebiotics (NPW, non-prebiotics with water) or pathogens (WRS, water with pathogen). However, the introduction of prebiotics (PRS) restores the stability of the network (Supplementary Fig. 3). Further exploration of community interactions was conducted at the genus level. Among all 491 identified genera, 474 genera were found to have negative or no correlations with the pathogen in relative abundance (Supplementary Table 7). Prebiotic application (PW and PRS) increased the abundance of microorganisms that were not positively associated with *R*. *solanacearum* compared to the water control and non-prebiotic metabolites application (Fig. 2g). These microorganisms, which are not positively correlated with *R*. *solanacearum*, may play an important role in maintaining plant health as commensal bacteria in the soil. Compared to the WW (only water), the abundance of microorganisms positively associated with *R*. *solanacearum* increased in the non-prebiotic treatment, while there was no significant change in the prebiotic treatment (PW and PRS) (Supplementary Fig. 4).

### In-vitro and in-situ investigation of prebiotic impacts on potential biocontrol mechanisms

To investigate potential mechanisms behind the prebiotic-induced control of the pathogen (Fig. 2a), we first tested the hypothesis that prebiotic application could directly stimulate the plant’s defensive mechanisms, thereby reducing the disease incidence. However, the results of our experiments conducted under limited bacterial conditions did not support this hypothesis, as adding prebiotics did not decrease disease incidence (Supplementary Fig. 5). We subsequently tested a second hypothesis that these compounds favor the growth of the commensal microbes in the rhizosphere, but not the pathogen, thereby indirectly reducing the disease incidence in tomato plants. This hypothesis was based on our observation of a negative correlation between the pathogen and the total bacterial abundances in the greenhouse experiment (Fig. 2c).

**Fig. 3:**
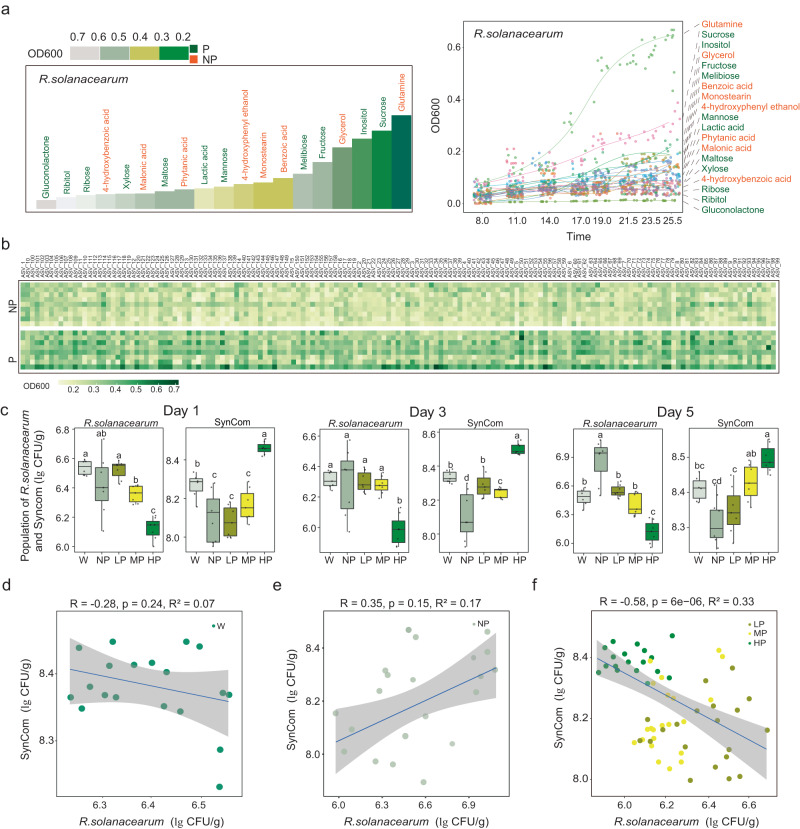
The utilization of commensal bacteria and *R. solanacearum* with different metabolites as carbon source. **a** *R. solanacearum* utilization of different carbon sources, with darker colors indicating better utilization. Nineteen rhizosphere metabolites were investigated, and those enriched in healthy tomato rhizosphere were highlighted in green, while the rest were enriched in diseased tomato rhizosphere; **b** Utilization of 17 carbon sources by 158 strains of bacteria isolated from rhizosphere soil, with darker colors representing better utilization. From top to bottom, mixture of eight non-prebiotics, 4−hydroxybenzoic acid, 4−hydroxyphenyl ethanol, benzoic acid, phytanic acid, glutamine, glycerol, malonic acid, monostearin, ribitol, gluconolactone, lactic acid, maltose, ribose, xylose, mannose and mixture of seven prebiotics was showed in each row; **c** Impacts of various concentrations of the metabolite mixture on the growth of *R. solanacearum* and the SynCom (158 isolated bacteria) over a period of 5 days. Horizontal bars within boxes represent the median. The tops and bottoms of boxes represent 75th and 25th quartiles, respectively. The upper and lower whiskers represent the range of non-outlier data values. Outliers were plotted as individual points. Different lowercase letters indicated significant differences among respective groups based on two-sided tests for multiple comparisons by Turkey HSD corrections (*t*-test, adjusted *p* < 0.05, *n* = 7 biologically independent samples); **d**–**f** Correlations among the abundance of the SynCom and *R. solanacearum* under different treatments. The solid lines represent regression lines and transparent areas represent 95% confidence interval. Two-sided t-test was used to test the significance of regression at 5% significance level. P prebiotics, NP non-prebiotics, LP: 1 µM prebiotic, MP: 100 µM prebiotic, HP: 10 mM prebiotic, NP: 10 mM non-prebiotic, W: sterile water.

The metabolic ability of *R. solanacearum* was tested with prebiotics and non-prebiotics, respectively. It was found that among the 11 prebiotics tested, sucrose, fructose, melibiose, and inositol were better utilized by *R. solanacearum* compared to the other seven prebiotics (gluconolactone, inositol, lactic acid, maltose, mannose, ribose, and xylose). In contrast, *R. solanacearum* could partially utilize non-prebiotics (Fig. 3a). To further test the utilization efficiency of tomato rhizosphere microorganisms for prebiotic metabolites (gluconolactone, inositol, lactic acid, maltose, mannose, ribose, and xylose) and non-prebiotic metabolites (benzoic acid, malonic acid, 4-hydroxybenzoic acid, 4-hydroxyphenyl ethanol, glycerol, glutamine, monostearin, and phytanic acid). We first isolated and purified tomato rhizosphere microorganisms by dilution coating plate method and obtained 158 strains, which were used to determine the growth curve under metabolite intervention (Fig. 3b). The results demonstrated that tomato rhizosphere microorganisms exhibited a higher utilization efficiency for prebiotics than non-prebiotics (benzoic acid, malonic acid, 4-hydroxybenzoic acid, phytanic acid, 4-hydroxyphenyl ethanol, glycerol, glutamine, and monostearin) (Fig. 3b). This suggested that prebiotics may have a greater potential to support the growth of microbes in the rhizosphere and contribute to plant health.

To validate our findings in-situ, a pot experiment was conducted, where the 158 selected bacterial strains and *R. solanacearum* were applied to soils along with a mixture of the non-prebiotics (1 μmol g^−1^ soil) or prebiotic at three concentration levels (0.1 nmol g^−1^, 10 nmol g^−1^ soil and 1 μmol g^−1^ soil). On the 1st, 3rd, and 5th day, soil with the inoculated *R. solanacearum* and co-cultures of the bacterial isolates were spread onto TSA and SMSA plates to count their cell numbers. Consistently, we found that amendments of the prebiotic mixture at HP (10 mM) effectively reduced cell numbers of the pathogen (Fig. 3c and Supplementary Table 8). The prebiotic mix did not impact the cell numbers of total bacterial strains at LP (1 µM prebiotics mix) and MP (100 µM prebiotics mix), but significantly increased in response to the HP (10 mM prebiotics mix) (Fig. 3c and Supplementary Table 8). The trend of changes in cell numbers was consistent over time (Fig. 3c). Furthermore, univariate linear regression analysis showed that the abundance of the pathogen was negatively correlated with that of the isolates in the LP (1 µM prebiotics mix), MP (100 µM prebiotics mix), and HP (10 mM prebiotics mix) treatments (Fig. 3f, R = −0.58; *p* < 0.05; R^2^ = 0.33), while the relationship in W (water) and NP (10 mM non-prebiotics mix) treatment was not significant (Fig. 3d, e).

### The metabolite mixture consistently suppressed bacterial wilt disease suppression in *Solanaceae* crops

**Fig. 4:**
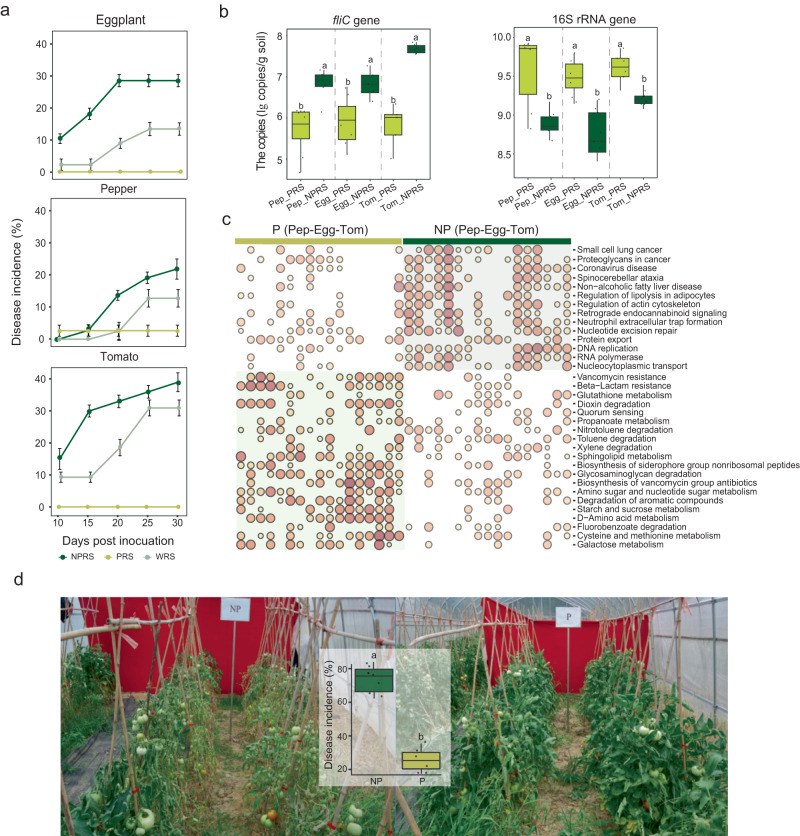
The effects of prebiotics on disease incidence and rhizosphere microbiome of three *Solanaceous* crops. **a** Development of bacterial wilt disease in eggplant, pepper and tomato plants 30 days after soils were amended with NPRS, PRS, or WRS. All data are presented as mean ± standard error of the mean (SEM) in line graphs (*n* = 6 biologically independent samples); **b** Copy numbers of *fliC* and 16S rRNA genes among rhizosphere samples of different treatments. Horizontal bars within boxes represent the median. The tops and bottoms of boxes represent 75th and 25th quartiles, respectively. The upper and lower whiskers represent the range of non-outlier data values. Outliers were plotted as individual points. Different lowercase letters indicated significant differences among respective groups based on two-sided tests for multiple comparisons by Turkey HSD corrections (*t*-test, adjusted *p* < 0.05, *n* = 8 biologically independent samples); **c** Bubble plot showed the enriched pathways of metagenomic data from rhizosphere of three *Solanaceae* crops after metabolites application; **d** The tomato field experiment used to test the biocontrol effect of metabolites. Horizontal bars within boxes represent the median. The tops and bottoms of boxes represent 75th and 25th quartiles, respectively. The upper and lower whiskers represent the range of non-outlier data values. Outliers were plotted as individual points. Different lowercase letters indicated significant differences among respective groups based on two-sided tests for multiple comparisons by Turkey HSD corrections (*t*-test, adjusted *p* < 0.05, *n* = 8 biologically independent samples). WRS water with pathogen, PRS prebiotics with pathogen, NPRS non-prebiotics with pathogen, Pep_NPRS pepper, non-prebiotic treatment, Pep_PRS pepper, prebiotic treatment, Egg_NPRS eggplant, non-prebiotic treatment, Egg_PRS eggplant, prebiotic treatment, Tom_NPRS tomato, non-prebiotic treatment, Tom_PRS tomato, prebiotic treatment, P prebiotics, NP non-prebiotics.

Since prebiotics applied at 1 μmol g^−1^ soil significantly reduced the pathogen population and increased the other bacterial populations, we further tested the impacts of the 1 μmol g^−1^ soil, seven metabolites (gluconolactone, inositol, lactic acid, maltose, mannose, ribose, and xylose) mixture on disease suppression in a greenhouse experiment. To examine whether our prebiotic cocktail was able to provide pathogen protection on other crops besides tomato, we extended our experiment to other *Solanaceae* crops; using pepper, eggplant, and a second tomato cultivar. The metabolite mixture significantly reduced bacterial wilt disease incidence in all three crops 30 days after inoculation of *R. solanacearum* and at a dose of 1 μmol g^−1^ soil of metabolite mixture (Fig. 4a and Supplementary Table 9). The abundance of total bacteria was significantly increased, and the abundance of pathogen was significantly decreased in all three species of *Solanaceae* crops after adding prebiotics compared to non-prebiotics (Fig. 4b and Supplementary Table 10). The bacterial wilt incidence of eggplant, pepper, and tomato increased by 16.1%, 9.54%, and 8.4%, respectively, after the addition of non-prebiotics compared to the control, while prebiotic application completely controlled disease occurrence across the 30-day experiment (Fig. 4a and Supplementary Table 9). The disease severity/incidence for pepper in the NPRS (non-prebiotics with pathogen) treatment was significantly lower than the other two crops (*p* < 0.05), but still, the prebiotics exhibited significant disease suppression, with a 2.7% disease incidence in the PRS (prebiotics with pathogen) treatment, compared with 13.3% in the WRS (water with pathogen) treatment and 22.8% in NPRS (non-prebiotics with pathogen) treatment. Additionally, we tested the biocontrol effect of prebiotics in tomato fields that had undergone continuous cropping, leading to high disease incidence (>50%). As shown in Fig. 4d, the disease incidence in tomatoes with added prebiotics was 25.0%, while with added non-prebiotics was 77.2%. In comparison, the disease incidence in the control treatment was 51%. Quantitative results showed that adding prebiotics significantly reduced the number of *R. solanacearum* (Supplementary Fig. 6).

**Fig. 5:**
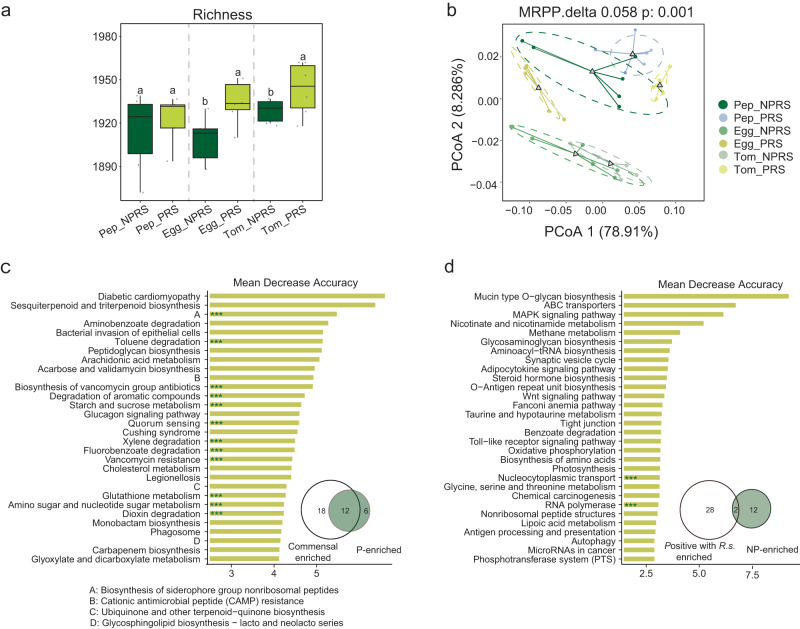
Importance ranking of microbial functional features in random forest analysis. **a** *Alpha* diversity of the rhizosphere soil bacterial communities across different treatments. Richness was calculated using the normalized ASV table. Horizontal bars within boxes represent the median. The tops and bottoms of boxes represent 75th and 25th quartiles, respectively. The upper and lower whiskers represent the range of non-outlier data values. Outliers were plotted as individual points. Different lowercase letters indicated significant differences among respective groups based on two-sided tests by Wilcoxon rank-sum test followed by Dunn’s multiple comparison test (adjusted *p* < 0.05, *n* = 9 biologically independent samples); **b** Principal coordinate analysis (PCoA) with Bray–Curtis dissimilarity performed on the functional profile of the eggplant, pepper and tomato microbiome at different treatment. The *p*-values were evaluated via the MRPP test; **c** Ranking of functional pathways driven by commensal microbes (not positively correlated with *R. solanacearum*) in prebiotic treatment; d: Ranking of functional pathways driven by microbes (positively correlated with *R. solanacearum*) in non-prebiotic treatment. *** indicated that the pathway was presented simultaneously in Fig. 5c, and Fig. 4c, or in Fig. 5d and Fig. 4c. Pep_NPRS pepper, non-prebiotic treatment, Pep_PRS pepper, prebiotic treatment, Egg_NPRS eggplant, non-prebiotic treatment, Egg_PRS eggplant, prebiotic treatment, Tom_NPRS tomato, non-prebiotic treatment.

To resolve the functional basis of prebiotics in assisting plant disease resistance, we used a bird shot macro-genomic assay for rhizosphere soils of three crops inoculated with prebiotic and non-prebiotic in pot experiment. The data volume for each sample was greater than 30 GB, yielding 1020 G data, and a total of 18 million predicted genes. Results revealed that prebiotics application increased rhizosphere microbial community diversity (richness) in three crops, with two crops (tomato and eggplant) significantly increased (*p* < 0.05; Dunn’s) (Fig. 5a). Ranking analysis of all genes by PCoA showed that the prebiotic treatment was significantly (*p* < 0.05; MRPP test) (Fig. 5b) different when compared to non-prebiotic treatment.

The GSVA (gene set variation analysis) enrichment analysis showed that prebiotic inputs significantly impacted glycosaminoglycan metabolism pathways, including galactose metabolism, starch and sucrose metabolism, and glycosaminoglycan degradation. Additionally, the degradation pathway of autotoxin substances, such as toluene degradation, xylene degradation, and fluorobenzoate degradation, was also enriched in the prebiotic treatments (Fig. 4c). To further investigate the diversity of microorganisms contributed to the metabolism pathways of prebiotics and non-prebiotics, we performed *alpha* diversity analysis. It was found that the diversity of microorganisms involved in the metabolism pathways of prebiotics was significantly higher than that of non-prebiotics (Supplementary Fig. 7).

### Commensal microbes contributed to functional changes in rhizosphere soil microbial communities with prebiotics application

We found that adding prebiotics increased the abundance of commensal microbes (not positively with *R*. *solanacearum*) (Fig. 2g). To investigate the relationship between the functional changes with the application of prebiotics and commensal microbes, the random forest method was used to assess the contribution of commensal microbes to the function of the community. Among the top 30 functional pathways, twelve pathways identified by random forest method (including Vancomvcin resistance, Degradation of aromatic compounds, Dioxin decradation, Quorum sensing, Toluene decradation, Xylene degradation, Biosynthesis of siderophore group non-ribosomal peptides, Biosynthesis of vancomycin group antibiotics, Amino sugar and nucleotide sugar metabolism, Glutathione metabolism, Starch and sucrose metabolism, Fluorobenzoate degradation) (Fig. 5c) were mainly contributed by commensal microbes and found to be enriched in prebiotic application (Fig. 4c). However, only two pathways were primarily driven by microbes positively correlated with by microbes positively correlated with *R*. *solanacearum* (Fig. 5d) and enriched in the treatment of non-prebiotic application (Fig. 4c).

## Discussion

In this study, we identified a set of key rhizosphere metabolites which were associated with healthy tomato plants under *R. solanacearum* pressure. Importantly, these metabolites significantly reduced bacterial wilt disease incidence of tomato, pepper and eggplant when applied as a 10 mM mixture to soil infected with *R. solanacearum*. Our results provide mechanistic knowledge that rhizosphere metabolites can be employed to promote the growth and diversity of commensal microbes in the plant rhizosphere, which resulted in effective pathogen suppression in plants and soils. These findings highlight the significant potential for the rhizosphere metabolites to be developed as novel prebiotics to control bacterial wilt diseases in important agricultural crops. Although using prebiotics in botany and agriculture is a relatively new concept, previous studies have provided some supporting evidence. Organic amendments typically serve as “prebiotics” and have been reported to regulate rhizosphere soil microbial functions^23^. In particular, rhizosphere metabolites have been found to play a central role in modulating plant-microbe interactions^24^. For example, anti-microbial metabolites of *Arabidopsis thaliana* were shown to confer tissue-specific resistance to a wide range of bacterial pathogens^25^. Similarly, specific organic acids were linked to plant performance and increased *Pseudomonas* abundance and colonization of tomato roots^26^. Lastly, our previous results revealed that amino acids (isoleucine, leucine, methionine, proline, tryptophan, and ornithine) and fatty acids mediated microbially induced plant defense against foliar pathogens^18^. These lines of evidence, among others, point to rhizosphere metabolites as promising compounds for the development of “rhizosphere prebiotics”. In this study, we further explored the function of prebiotics that decreased pathogen invasion by demonstrating their stimulation of commensal rhizosphere microbes.

A non-targeted metabolomic approach used in this study detected eleven metabolites enriched in the rhizosphere of healthy tomato plants, with the majority being small-molecular weight sugars. Small molecular weight carbohydrates, such as arabinose, glucose, galactose, fructose, sucrose, pentose, rhamnose, raffinose and xylose, have been previously reported to alleviate plant stresses^27^. For instance, different plant species produced xylose in different concentrations in the rhizosphere. A higher amount of xylose in the rhizosphere stimulated a plant signaling pathway that triggers the biocontrol activity of *Bacillus velezensis*^28,29^. *Myo*-inositol compounds were higher in concentration in tomato genotypes with salinity tolerance^30^, and have also been demonstrated to play an important role in *R. solanacearum* inhibition in *Tamarindus indica*^31^. This is further supported by the upregulation of the plant genes encoding β−1,3-glucanase, chitinase and cell wall invertase by the infection of *Ralstonia solanacearum* in tomato^32^.

Previous studies on prebiotics have been focused on metabolites which recruit beneficial microbes such as *Bacillus*, *Streptomyces* and *Pseudomonas*^33,34^. In fact, the commensal (neutral) microbes, which do not display obvious beneficial effects on plants, are often present in larger abundances and with greater diversity than those deemed as beneficial. The commensal organisms can directly influence plant health. Our study provides a different mechanism of metabolite-derived pathogen suppression than many previous reports where plants actively called for beneficial microbes to suppress specific pathogens^35,36^. The commensal microorganisms referred in this study are the abundant soil microorganisms that do not show significantly positive correlations with *R. solanacearum* (Fig. 2g), and those microbial abundances and diversity were stimulated by the application of the prebiotics (Fig. 2d–f). This suggests that commensal microbes, stimulated by prebiotics, can outcompete pathogens in occupying niches, thus reducing the occurrence of infections and diseases^5,37^.

Recently, synthetic microbial communities (SynCom) have been regarded as a useful tool to understand the interaction between microbes and plants, and many studies have provided a guideline in constructing microbial communities^38^. We used the 158 isolates as a commensal microbial SynCom that encompassed the main phylogenic groups in soil with prebiotics to study their interactions with the pathogen and host plants^39^. Though this SynCom did not fully represent the whole microbial diversity of the rhizosphere soils, it provided direct evidence that the prebiotic-mediated effects were driven by the enrichment of commensal bacteria.

Here, we utilized a metagenomic approach to show the biosynthesis of siderophore group non-ribosomal peptides enriched by commensal microorganisms (Fig. 5c). In addition, we discovered several important functions driven by commensal microorganisms, including biosynthesis of vancomycin group antibiotics, degradation of aromatic compounds, quorum sensing, fluorobenzoate degradation, vancomycin resistance, and dioxin degradation. Some of these functions are related to the breakdown of harmful substances in the soil, which can weaken plant immunity and promote disease. For example, the degradation of aromatic compounds, fluorobenzoates, and dioxins^40^.

The efficacy of this prebiotics on other Solanaceae crops further supports the potential of disease control through modulation of the soil microbiome (Figs. 4a and 5a). Metagenomic analysis of rhizosphere soils revealed that prebiotics enhances carbon metabolism-associated pathways such as galactose metabolism, starch and sucrose metabolism, and glycosaminoglycan degradation, along with a high level of microorganisms driving these functional enrichments (Fig. 4c and Supplementary Fig. 7). This suggests that prebiotics, as “food” for commensal microbes, are important in shaping microbial community structure and multifunctionality, thus reducing the ecological niche for pathogenic bacteria to survive^41,42^. Furthermore, prebiotics could enhance the degradation ability of root autotoxins, including toluene degradation, nitrotoluene degradation, xylene degradation, and degradation of aromatic compounds, demonstrating the ability of prebiotics to promote plant health by activating soil microorganisms to eliminate harmful root autotoxins^43^.

**Fig. 6:**
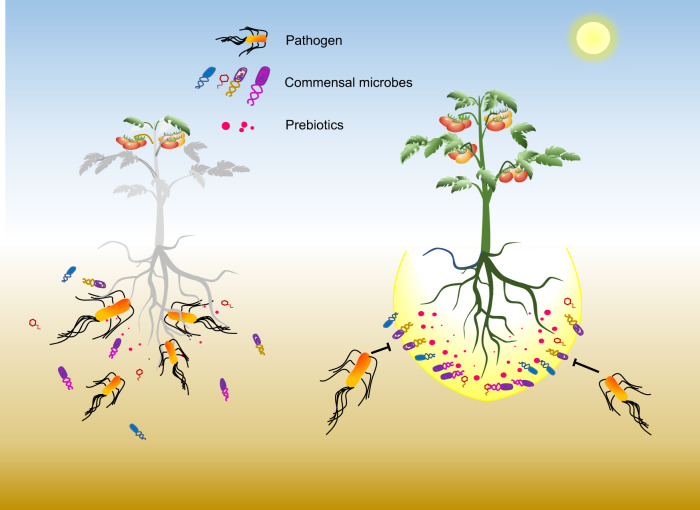
The conceptual figure illustrates the potential of prebiotics in alleviating bacterial wilt by recruiting commensal microbes. This figure illustrated that prebiotics investigated from rhizosphere can prevent plant disease by increasing the number of commensal microbes in the rhizosphere soils to antagonize pathogenic invasion in tomato and other plants.

Application of our metabolite mixture as prebiotics correlated strongly with changes in abundance and diversity of the native rhizosphere bacterial community. *Ralstonia solanacearum* infection also dramatically influenced the composition and diversity of rhizosphere microbiome. Notably, metabolites enhanced bacterial population size and diversity, whereas pathogen infection reduced both metrics. Furthermore, the metabolite mixture alleviated the negative impact of pathogen infection on the diversity of rhizosphere microbiome. Previous work demonstrated the effect of applied phages on a tomato rhizosphere microbiome, where the phages were selected to kill *R. solanacearum*^44^, and it is likely increased bacterial diversity was linked to the targeted reduction of pathogen abundance. In the current study, we stimulated a number of commensal bacteria and “targeted starvation” of pathogen. The results suggested that “prebiotics” used in our study could be a safe and environmentally friendly bioresource for controlling plant pathogenic bacteria. The increased bacterial diversity can be beneficial for the resistance of other pathogen species. Additionally, this biocontrol was effective in several crops, pepper, tomato, and eggplant and therefore is ideal for further biopesticide development. We revealed rhizosphere metabolites that could not be efficiently utilized by a targeted pathogen but could be consumed by a diverse set of commensal microbes can be used as prebiotics to support plant health and disease suppression via engineering soil microbiome (Fig. 6).

## Methods

### Growth, disease development, and rhizosphere soil collection from tomato plants

A field experiment was set up to examine the difference between the rhizosphere metabolomes and microbiomes of disease-free versus diseased tomato plants. Three hundred tomato seeds (*Solanum lycopersicum* cv. HeZuo 903 is a variety of tomato widely cultivated in the Jiangsu province and was provided by the Vegetable and Flower Research Institute of Nanjing) were soaked in 100 mL NaClO solution (0.75%, v/v) for 30 min, washed five times with sterile water, and pregerminated in sterile Petri dishes at 25 °C for 3 days. Approximately 240 germinated seeds were planted in a nursery substrate (commercially available from Huaian Agricultural Technology Development Ltd., Huaian, Jiangsu, China), with one seed sown per well (5 × 5 × 5 cm) of the seeding trays (50 wells per tray). The substrate and seeds were then incubated at 28 ± 3 °C with a 16 h/8 h (light/dark) cycle in the greenhouse and watered daily as needed to maintain soil moisture for three weeks. A total of 120 healthy seedlings were carefully transplanted to the field (31°43′ N, 118°46′ E) where 6 years of continuous cropping of tomato (HeZuo 903) had been conducted with two seasons per year. The average disease incidence for the past season was approximately 50%. All plant residues were removed at the end of each season. This region has a typical subtropical monsoon climate, with a mean annual temperature of 18 °C and an annual precipitation of 1416 mm. The soil is classified as an Ultisol, which is widely distributed throughout the subtropical areas of South China. Tomato seedlings were planted in a 2 × 5 m plot, with 0.5 m distance between plants in March, 2017. At the fruiting stage (10 weeks after transplanting), we observed that some plants displayed typical bacterial wilt disease symptoms; top leaves of the tomato plants appear dehydrated with wilting and drooping, but the wilting leaves remain green for some of the tomato plants. After this inventory, 12 healthy and 12 diseased plants were randomly selected across the experiment. The roots of these plants were collected together with the adhering soil and stored in bags on ice prior to transport to the laboratory. Then, three plants of the same health condition (diseased/healthy) were mixed as one biological replicate, thereby resulting in four composite biological replicates for each disease condition. Soil samples were then recovered according to previously described methods^45,46^ for microbiome and metabolite analyses. Briefly, soil loosely attached to the roots was shaken off and discarded, and root tissues with tightly associated soil were then cut into segments (~1 cm) under aseptic conditions. These root segments were washed with 200 mL sterile water and the resulting suspension was considered as the rhizosphere sample. Half the suspension was lyophilized to remove water for metabolite extraction. The rest of the suspension was centrifuged at 10,000 g at room temperature for 10 min, and the precipitate soil was stored at −20 °C prior to DNA extraction.

### Extraction of the rhizosphere soil metabolites

The metabolites from rhizosphere soil were extracted and analyzed using a modified version of the protocol described by ref. ^46^. Briefly, each soil sample was split into two equal parts (0.2 g each) in two 2 mL Eppendorf (EP) tubes, and 24 μL of adonitol (1 mg mL^−1^) was added to each tube as an internal standard. One part of the rhizosphere soil was homogenized in 0.5 mL methanol solution (V_methanol_: V_H2O_ = 3:1) using a ball mill at 45 Hz for 4 min and then ultrasonically treated 5 times for a period of 5 min each.

The soil was then centrifuged at 10,000 g at 4 °C for 15 min, and the supernatant (~0.4 mL) was transferred to a fresh EP tube. A second extraction was performed with 0.5 mL ethyl acetate using the same method as above, and the resultant extracts were combined (~0.8 mL in total). Then, 0.5 mL of ethyl acetate and 0.5 mL of methanol solution were applied to each soil sample at the end of the extraction procedure. A second portion of soil sample was extracted by another 0.5 mL ethyl acetate followed by 0.5 mL methanol solution (V_methanol_: V_H2O_ = 3:1) using the same method, resulting in an additional extract (~0.8 mL). After four extractions, each sample yielded 1.6 mL solution. The extractions were analyzed by BIOTREE technology Co. Ltd. (Shanghai, China) using a gas chromatograph (Agilent 7890) coupled with time-of-flight mass spectrometry (GC-TOF-MS) as per the manufacturer’s instructions. Raw data processing and analyses were performed as previously reported^47^. Briefly, Chroma TOF 4.3X software of the LECO Corporation and the LECO-Fiehn Rtx5 database were used for raw peaks exactions, data baseline filtering, calibration of the baseline, peak alignment, deconvolution analysis, peak identification and integration of the peak area^48^. Metabolites were identified using both mass spectrum and retention index matches.

We compared the relative abundance of rhizosphere metabolites of healthy versus diseased plants to identify compounds that were more abundant in the healthy and diseased plant samples. Using the general linear model (GLM) and random forest model, we identified eleven compounds (melibiose, sucrose, ribose, lactic acid, xylose, inositol, mannose, fructose, maltose, gluconolactone, and ribitol) with high relative abundance in healthy samples and eight compounds (benzoic acid, malonic acid, 4-hydroxybenzoic acid, phytanic acid, 4-hydroxyphenyl ethanol, glycerol, glutamine, and monostearin) with high relative abundance in diseased samples. These compounds were selected for further investigation.

### Impacts of the selected compounds on tomato disease tolerance and the rhizosphere microbiome

Tomato seeds (Hezuo 903) were sterilized and germinated on half strength Murashige and Skoog medium (MS, Haibo technology Co. Ltd. in Qingdao, China) supplemented with 1% sucrose for 5 days (16 h light, at 25 °C and 46% relative humidity; 8 h darkness, at 18 °C and 37% relative humidity). Plant seedlings were then transplanted in pots (length/width/height = 5 × 5 × 5 cm), each with 50 g soils and grown in a greenhouse under a 16 h photoperiod (120 μmol photons m^−2^s^−1^) at 23/20 °C day night^−1^ temperature.

A prebiotic cocktail was prepared with an equimolar proportion of all of the eleven metabolites described above, and its ability to impede the pathogen *R. solanacearum* was examined. The experimental design consisted of the following six treatments, (1) PRS: prebiotics and the pathogen *R. solanacearum*; (2) PW: prebiotics and water; (3)WRS: water and the pathogen; and (iv) WW: only water; (5) NPRS: non-prebiotics (eight metabolites enriched form diseased plants) and the pathogen *R. solanacearum*; (6) NPW: non-prebiotics and water. Each treatment was conducted using 108 plants in three replicates (36 plants per replicate). After an initial 4 weeks of plant growth, 5 mL of the prebiotic cocktail (each of the 11 compounds were mixed with an equal volume of a 10 mM solution) or water was applied to the soil once a week for 4 weeks as 4 doses of 1 µmol g^−1^ soil. The pathogen (5 × 10^8^ CFU per 50 g per plant) was inoculated to the soil after the first application of prebiotics. The plants were watered daily to maintain soil moisture during the whole period of the greenhouse experiment.

Plants with typical bacterial wilt symptoms (necrosis and drooping leaves) were considered as diseased plants, and disease incidence was quantified using the following formula: diseased plant number/ total plant number × 100%^49^. The plant disease incidence was recorded for each of the treatments throughout the experiment. To track microbial community responses to prebiotic application, rhizosphere soil samples were collected as described above from nine healthy plants without the bacterial wilt symptom appeared, and stored at −80°C prior to subsequent DNA extraction.

### Profiling the tomato rhizosphere microbiome

Total genomic DNA was extracted from 0.5 g rhizosphere soil using the PowerSoil DNA Isolation Kit (Qiagen, German) per the manufacturer’s instructions, with resulting DNA quantified using a NanoDrop spectrophotometer (ND2000, Thermo Scientific, DE, USA). The V4 region of the 16S rRNA gene was amplified by PCR using the primer pair 515 F (5′-GTGYCAGCMGCCGCGGTAA-3′) and 806 R (5′-GGACTACNVGGGTWTCTAAT-3′).

The 50 μL reaction mixtures contained 25 μL 2× Premix Taq (Takara Biotechnology, Dalian Co. Ltd., China), 1 μL each primer (10 μM), 3 μL DNA (20 ng/μL), and 20 μL of sterilized ultrapure water. The following cycles were used for PCR amplification with a Bio-Rad S1000 (Bio-Rad Laboratory, CA, USA): 95 °C for 5 min, then 30 cycles of 94 °C for 30 s, 52 °C for 30 s, and 72 °C for 30 s with a final extension at 72 °C for 10 min. DNA Marker (100–2000 bp; B500350 Sangon Biotech (Shanghai) Co., Ltd.) was used as DNA marker on a 1% agarose gel, and samples with clear bands between 290 and 310 bp were combined for sequencing. According to GeneTools analysis software (version 4.03.05.0, SynGene), PCR products were mixed at equal densities. A Gel Extraction Kit (Omega, USA) was used to purify the mixture.

According to the manufacturer’s instructions, sequencing libraries were prepared using the NEBNext® UltraTM DNA Library Prep Kit for Illumina® (New England Biolabs, USA). The library quality was assessed using a Qubit® 2.0 Fluorometer (Thermo Scientific) and an Agilent Bioanalyzer 2100. The library was sequenced on an Illumina Hiseq 2500 platform (Magigene, Guangdong). Based on the unique barcode of each sample, 250-bp paired-end reads were filtered using Trimmomatic (V0.33) to obtain clean, high-quality reads.

Total bacterial abundance and pathogen load in the rhizosphere were quantified using quantitative PCR (qPCR). Primers 347 F (5′-GGAGGCAGCAGTRRGGAAT-3′) and 531 R (5′-CTNYGTMTTACCGCGGCTGC-3′)^50^ were used for quantification of total soil bacterial density. The *fliC* gene (forward, 5′-GAACGCCAACGGTGCGAACT-3′; reverse, 5′-GGCGGCCTTCAGGGAGGTC-3′)^51^, encoding the flagella subunit, was the target used for *R. solanacearum* quantification^52^. All qPCR assays were performed using a StepOne Plus^TM^ Real-Time PCR System (ABI Co. Ltd, USA). Standard curves were generated using 10-fold serial dilutions of a plasmid containing the *fliC* gene from *R. solanacearum* and the amplified fragment region from the V4 region comes from the model strain *Bacillus subtilis*168. A serial dilution from 10^9^ to 10^3^ gene copies/μL of the *fliC* gene and V4 region amplification products was used as a standard, with an amplification efficiency of 103.6% and 98.6%, respectively. We then performed the qPCR assay with a 20 μL reaction mixture containing 10 μL of SYBR Premix Ex Taq (2×), 1 μL of each primer (10 μmol/L), 0.4 μL of ROX Reference Dye II, 1 μL of template DNA (20 ng/μL) and 6.6 μL of sterile water. Each DNA sample was analyzed in three replicates. By analyzing melt curves and electrophoresizing agarose gels, the specificity of the amplified fragments was confirmed. After calculating the copy number of each target fragment from the standard curves, the results were expressed as log10 values (copies/g soil). The thermal cycling profile included a first step at 95 °C (30 s) followed by 40 cycles of 95 °C for 5 s and 60 °C for 30 s, with a final extension at 72 °C for 5 min.

### Investigating potential interactions among prebiotics, resident soil isolates, and bacterial wilt suppression

#### Bacterial isolation from the tomato rhizosphere

In order to test the potential mechanism of prebiotic on commensal soil bacteria, we first isolated bacterial strains from the rhizosphere of the tomato plant. One half gram of lateral roots with the attached rhizosphere soil were washed with 1 mL sterile water in a 2 mL centrifuge tube by vertexing at 3200 rpm/min for 30 min. Then, 100 μL soil suspension was used for serial dilution using sterile water, and an aliquot of 100 μL of a 10^−6^ dilution was inoculated on a 1/3 strength TSA plates in triplicates and incubated at 28°C for 3 days in an incubator (Shanghai CIMO Medical Instrument Manufacturing Co., Ltd, China). A total of 1000 single colonies were picked from the plates and purified twice. To provide taxonomic identification of each isolate, purified cultures were subjected DNA extraction (B518255, Sangon Biotech, Shanghai), PCR amplification and amplicon sequencing, which were performed at Qingke Biotechnology (Nanjing, China) according to ref. ^53^. The obtained raw sequences were quality filtered and demultiplexed and taxonomy of the sequences was classified using the Greengenes 13.5 database (method detailed in the bioinformatics for 16S rDNA amplicon sequencing analyses of this study).

#### Effects of 11 metabolites on the growth of *R. solanacearum* and the isolated bacterial strains

The selected metabolites were tested for their effects on the growth of the pathogen and 158 bacterial strains. *R. solanacearum* and isolated strains were experimented with using NB (Nutrient Broth) and TSB (Tryptic Soy Broth) media, respectively. The media inoculated with the microbes were shaken at 170 rpm min^−1^, 28 °C for 24 h and cell density was diluted to an OD 0.01 with sterilized water. The bacterial suspension (2 µL) was then inoculated into a sterile 96-well plate (Costar, Coring Incorporated, USA) with 200 µL inorganic salt medium ((NH_4_)_2_SO_4_ 2.0 g L^−1^, MgSO_4_·7H_2_O 0.2 g L^−1^, CaCl_2_·2H_2_O 0.01 g L^−1^, FeSO_4_·7H_2_O 0.001 g L^−1^, Na_2_HPO_4_·12H_2_O 1.5 gL^−1^, KH_2_PO_4_ 1.5 g L^−1^). A final concentration of 10 mM for 11 healthy metabolites and 8 diseased metabolites was applied as carbon sources to the bacterial culture in the respective wells of the plates. The same volume of the bacterial suspension but amended with equal amount of sterile water was used as control. Hereafter, all the 96-well plates were incubated at 28 °C at 170 rpm for 24 h and the absorbance of the culture at OD600 was measured at specified time intervals using SpectreMax M5 (Molecular Devices, USA).

#### Co-culture of selected bacteria and pathogen to test the mechanism of prebiotic mimic the rhizosphere condition

By examining carbon source utilization by *R. solanacearum*, we eliminated four metabolites (sucrose, inositol, fructose, and melibiose) from an initial pool of eleven that exhibited improved utilization, and the remaining seven metabolites (ribose, lactic acid, xylose, mannose, maltose, gluconolactone and ribitol) were designated as candidates for further experimentation as potential prebiotics. To test the potential mechanism of prebiotic, we set up a precise experiment by reintroducing a SynCom with the above bacteria into the sterile soil. In this experiment, 150 g of soil were placed in tissue culture vessels and autoclaved three times to deactivate all soil microbes. The efficiency of the sterilization process was confirmed by plating samples on LB agar, which resulted in no colony growth. Three replicates of 5-day-old tomato seedlings (Micro-TOM) grown under sterile conditions were transferred into the sterilized soil in the tissue culture vessels. Then, to examine how application concentration impacted the effects of the prebiotic cocktail, we used three concentrations (1 µM, 100 µM, and 10 mM) of combined seven healthy metabolites and performed five treatments: (1) LP, 1 µM compounds mix, (2) MP, 100 µM compounds mix, (3) HP, 10 mM compounds mix was, and (4) W, and (5) NP 10 mM non-prebiotics mix (benzoic acid, malonic acid, 4-hydroxybenzoic acid, phytanic acid, 4-hydroxyphenyl ethanol, glycerol, glutamine, and monostearin), sterile water. Fifteen milliliters of the solution for each treatment were added once to soils 1-month after seedling transfer. All bacterial strains were propagated using 50 mL shake flask (170 rpm) in TSB medium, 28 °C for 24 h and cell density was diluted to an OD 0.01 (approximately ~10^7^ CFU/mL) with sterilized water. Subsequently, an equal proportion of each bacterial strain was mixed. The final concentration was adjusted to 1 × 10^8^ CFU/mL. Then, a 15 mL of mixture of the 158 isolated bacterial strains were added. Each treatment contained 144 plants.

The rhizosphere soils were collected on the 1st, 3rd, and 5th day post inoculation and the soil bacterial abundance was enumerated by serial dilutions and plate counts. At each timepoint, 40 plants were harvested and combined into 5 replicates. TSA plates were used for counting the total population of added 158 bacterial strains and semi-selective medium of South Africa (SMSA) plates were used for the pathogen counting^54^. All plates were cultured at 28 °C for 48 h in a thermostatic incubator (Shanghai CIMO Medical Instrument Manufacturing Co., Ltd, China).

### Effects of metabolites on plant health under sterile conditions

To test the effect of these metabolites directly on the plant defense, we counted the disease incidence on tomatoes after irrigation with prebiotics and pathogen under sterile conditions. In this experiment, 150 g of soil were placed in tissue culture vessels and autoclaved three times to deactivate all soil microbes. The efficiency of the sterilization process was confirmed by plating samples on LB agar, which resulted in no colony growth. After 1 month of growth of sterile seedlings, 15 ml of the prebiotic was irrigated into the soil. The seedlings receiving equal amounts of sterilized water and diseased metabolites were set up as negative control and positive control, respectively. Each treatment contained 108 plants. Then, the pathogen *R. solanacearum* was added to the soil at a final density of 1 × 10^7^ CFU g^−1^/soil. The disease incidence was recorded after plants were cultured in the above conditions for 60 days.

### Impacts of prebiotics on bacterial wilt disease incidence of three Solanaceae crops

Tomato (*Solanum lycopersicum*. cv. Micro-Tom), pepper (*Capsicum annum* L. cv. Yinchuan Cavel) and eggplant (*Solanum melongena* L. cv. Chunqiumoqie), which are all susceptible to bacterial wilt disease, were used to validate the biocontrol effects of the prebiotics. All seeds were first sterilized as described above, and planted in pots (length × width × height = 4 × 4 × 12 cm) with 150 g soil (the same soil as above). Three forma specialists of pathogenetic *R. solanacearum* strains for tomato, pepper and eggplant were cultured from glycerol stocks from −80 °C lab glycerol stocks and used in this experiment. Three treatments were set up for each crop: WRS (water with pathogen), PRS (10 mM prebiotics with pathogen), and NPRS (10 mM non-prebiotics with pathogen). The 10 mM metabolite concentration used in this assay was based on the results from co-culture assay in rhizosphere soil. Each treatment contained 108 plants in three blocks (36 plants per block). The application of prebiotics and the pathogen were as described above. Briefly, after 1 month of plant growth, 15 mL of a solution prebiotics or non-prebiotics, or water was drenched in soil. The solution of metabolites was applied to soils once every week at the same dosage for 4 weeks. The pathogen (1 × 10^8^ CFU mL^−1^) was inoculated to the soil after the first application of metabolites. Bacterial wilt disease incidence was recorded every 5 days throughout the experiment.

Rhizosphere soil samples collected from the three Solanaceae crops were used for shotgun metagenomic sequencing. Library preparation was performed in according to the Illumina, standard protocol. Briefly, DNA was fractionated by ultrasound, pooled libraries containing equimolar amounts of barcoded 350–500 bp fragments were prepared, and 150 bp paired end fragments were sequenced by Illumina Noveseq 6000. The raw metagenome sequence data (1020 Gb) were trimmed, filtered, assembled by MEGAHIT and contigs longer than 300 bp were used for further gene prediction and annotation. Open reading frames (ORFs) from assembled metagenomes were predicted using MetaGeneMark. The predicted ORFs with lengths longer than 100 bp were translated to construct a non-redundant gene catalog with criteria of 95% sequence identity and 90% coverage, and gene abundance in each sample was normalized into reads per kilobase million counts.

For taxonomic annotations, representative sequences in the gene catalog were searched against the non-redundant protein database of NCBI with an e-value cutoff of 1e^−5^ using DIAMOND and the lowest common ancestor method was applied to estimate the assignment of genes to specific taxa. For functional annotations, the Kyoto Encyclopedia of Genes and Genomes (KEGG) annotation were conducted with an e-value cutoff of 1e^−5^.

### Effects of prebiotics on tomato bacterial wilt disease incidence in field

To verify whether prebiotics can function effectively in the complex environment of the field, we experimented in the field with initially healthy and diseased tomato samples. Two treatments were set as follows: (1) NP: 10 mM non-prebiotics mix (benzoic acid, malonic acid, 4-hydroxybenzoic acid, phytanic acid, 4-hydroxyphenyl ethanol, glycerol, glutamine, and monostearin); (2) P: 10 mM prebiotics mix (ribose, lactic acid, xylose, mannose, maltose, gluconolactone and ribitol). Each treatment consisted of 90 tomato seedlings (3 replicates, and 30 plants per replicate).

The soil properties, seedling cultivation, and preparation of the metabolite solution were as previously described. The metabolite mixture was added every 2 weeks, with each seedling irrigated with 200 mL for 2 months. Disease incidence was recorded during the fruiting stage of the tomato, and rhizosphere soil was collected for qPCR analysis of pathogen and total bacteria. The collection method for rhizosphere soil and DNA extraction were as previously described. After the completion of the experiment, eight tomato plants were selected from each treatment for rhizosphere soil collection.

### Bioinformatics

Usearch (V. 10.1) and vsearch (V. 0.6.3) were used to process the sequencing data. First, the “vsearch --fastq_mergepairs” script was used to merge paired-end sequences; the “vsearch --fastx_filter” script was used to cut primers; the “vsearch --derep_fulllength” script was used for find unique sequence reads; the “usearch -unoise3” script was used to generated ASVs; the “vsearch --usearch_global” script was used to create an ASV table; and the “vsearch --sintax” script and RDP taxonomic database were together used for annotation of representative sequences. A normalized number of sequences was randomly extracted from each sample in order to calculate *alpha* diversity indices that were estimated with the vegan R package^55^.

For taxonomic annotations, representative sequences in the gene catalog were searched against the non-redundant protein database of NCBI with an e-value cutoff of 1e^−5^ using DIAMOND and the lowest common ancestor method was applied to estimate the assignment of genes to specific taxa. For functional annotations, the Kyoto Encyclopedia of Genes and Genomes (KEGG) annotation were conducted with an e-value cutoff of 1e^−5^.

### Statistical analyses

All statistical analyses were performed using the R 4.0 software environment, unless specified otherwise. For the differential analysis, the data were tested for normality by using the Shapiro-Wilk test of normality and for homogeneity of variances by using Levene’s test for homogeneity of variances.

Before the calculation of *beta* diversity, metabolites were standardized to relative abundance and Bray–Curtis similarity matrices were prepared using the “vegan” package (Version: 2.5-7)^56^. PERMANOVA (Adonis, transformed data by Bray–Curtis, permutation = 999) was used to determine if *beta* diversity significantly differed among treatments or plant disease states and principal coordinate analysis (PCA) plots were generated based on Bray–Curtis similarity matrices using the “ggplot2” package^57^ (Version: 3.3.5). For screening rhizosphere metabolites for the development of prebiotic, the general linear model (GLM) was firstly used to identify differentially expressed metabolites. The R package “mvabund”^58^ was used to fit the model. The significance threshold was set as adjusted *p* < 0.05. The *p*-values were corrected by the step-down resampling procedure for multiple comparisons. Then, metabolites were used to constructed random forest model for seek the metabolites could be well distinguished healthy/diseased. The “randomForest” package was used to develop random forest models. We first used the importance () function to rank individual metabolites on the basis of their contribution to the accuracy of the models. We then used the rfcv() function to perform a tenfold cross-validation that evaluated model performance as a function of the number of discriminant metabolites included in the model. We used similar statistical tools to contrast microbiomes across samples based on the 16S rRNA gene ASV table generated herein. Nonparametric t-tests were used for detection of significant differences in bacterial/ archaeal community diversity/richness based on Shannon diversity, Pielou evenness, and the Chao1 index. Before the calculation of *beta* diversity, relative abundances were used to standardize the ASV profiles. Bray–Curtis distance matrices were prepared using the “vegan” R package. PERMANOVA (Adonis, transformed data by Bray–Curtis, permutation = 999) was used to test if the *beta* diversity differed among treatments and principal coordinate analysis (PCoA) plots were generated according to Bray–Curtis similarity matrices created using the R package “ggplot2”^57^. Network analysis was performed using R package “ggClusterNet”^59^. To reveal microbial taxa that were more abundant in the metabolite treated samples, linear discriminant analysis (LDA) and effect size (LEfSe) analyses were performed with the “MASS” packages (Version: 7.3-54).

Linear regression was performed using R and the R2 (R-squared) and *p*-values were recorded with the function “lm” from the “stats” package. The “ggpubr” R package (https://CRAN.R-project.org/package=ggpubr) was used to produce correlation plots. If not specially specified, all plots were created with the “ggplot2” package in R-Studio.

### Reporting summary

Further information on research design is available in the Nature Portfolio Reporting Summary linked to this article.

## Supplementary information

Supplementary Information

Reporting Summary

Description of Additional Supplementary Files

Supplementary data 1

## Data Availability

All data required to reproduce the results are available in the Figshare database (10.6084/m9.figshare.23254319)^60^. Raw sequence data obtained in this study have been deposited in Genome Sequence Archive in the BIG Data Center, Chinese Academy of Sciences under accession codes CRA005139. Source data are provided with this paper.

## Source data

Source data

## Code availability

All codes used in this study are available on GitHub (https://github.com/taowenmicro/TaoWen.et.al.tomato.2022) and Zendo^61^.
