# Supplementary material for: Tapping the rhizosphere metabolites for the prebiotic control of soil-borne bacterial wilt disease
Source: Nat Commun. 2023 Jul 26;14:4497. doi: 10.1038/s41467-023-40184-2 (PMC10372070; doi:10.1038/s41467-023-40184-2)
Supplement: Supplementary file 1 — Supplementary Information [file 41467_2023_40184_MOESM1_ESM.pdf]

Supplementary Materials for

Tapping the rhizosphere metabolites for the prebiotic control of soil-borne bacterial wilt disease

Authors: Tao Wen [1, 2, a], Penghao Xie [1,a], Hongwei Liu[3], Ting Liu[1], Mengli Zhao[1], Shengdie Yang[1], Guoqing Niu[1], Lauren Hale[4], Brajesh K.Singh[2], George A. Kowalchuk[5], Qirong Shen[1], Jun Yuan [1,*]

Affiliations:

[1]Jiangsu Provincial Key Lab for Organic Solid Waste Utilization, Jiangsu Collaborative Innovation Center for Solid Organic Wastes, Educational Ministry Engineering Center of Resource-saving fertilizers, Nanjing Agricultural University, Nanjing 210095, China

[2]Key Laboratory of Green Intelligent Fertilizer Innovation, MARD, Sinong Bio-organic Fertilizer Institute, Nanjing 210000, China

[3]Hawkesbury Institute for the Environment, Western Sydney University, Penrith, NSW 2753, Australia

[4]USDA, Agricultural Research Service, San Joaquin Valley Agricultural Sciences Center, Parlier, California

[5]Ecology and Biodiversity Group, Department of Biology, Institute of Environmental Biology, Utrecht University, Padualaan 8, 3584 CH Utrecht, The Netherlands

[a] These authors have contributed equally to this work

*Corresponding author. Email: junyuan@njau.edu.cn

Supplementary Figure 1 Principal Component Analysis (PCA) ordination summarizing the differences of the rhizosphere metabolites between healthy and diseased samples. R and P values were calculated via two-way Adonis ($p < 0.05$) with 999 permutations. The obtained p value was corrected for multiple comparisons using Turkey HSD.

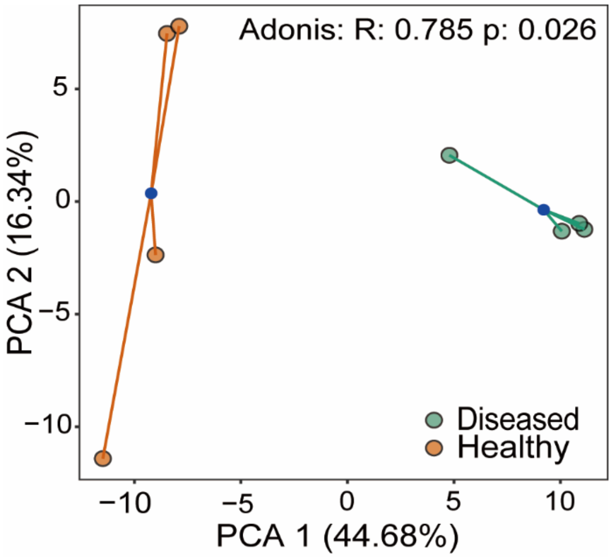

Supplementary Figure 2 The relative abundance of *Ralstonia solanacearum* in plants with different treatments. Horizontal bars within boxes represent the median. The tops and bottoms of boxes represent 75th and 25th quartiles, respectively. The upper and lower whiskers represent the range of non-outlier data values. Outliers were plotted as individual points. Outliers were plotted as individual points. Different lowercase letters indicated significant differences among respective groups based on two-sided tests by Wilcoxon rank-sum test followed by Dunn's multiple comparison test (adjusted $p < 0.05$, n = 6 biologically independent samples). WRS: water with pathogen; PRS: prebiotics with pathogen; NPRS: non-prebiotics with pathogen; PW: prebiotics with water; NPW: non-prebiotics with water; WW: only water.

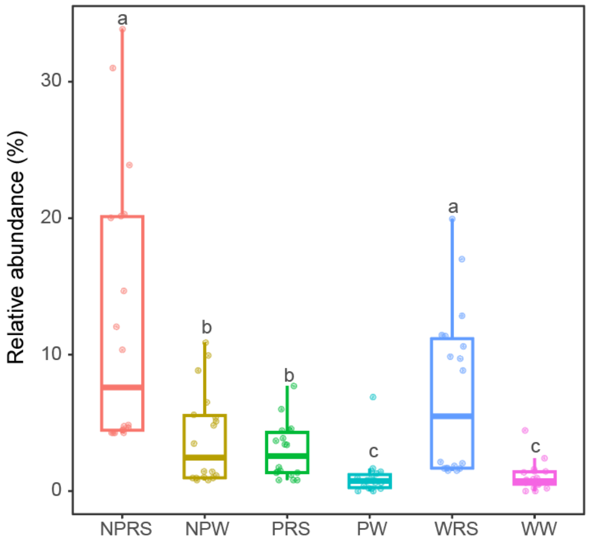

Supplementary Figure 3 The natural connectivity of microbial network. Point represent the average of data. Error bars represent standard errors (n = 6 biologically independent samples). NPW: non-prebitic + water, NPRS: non-prebitic + *R. solanacearum*, WW: water + water, PW: prebiotics + water, PRS: prebitic + *R. solanacearum*, WRS: water + *R. solanacearum*.

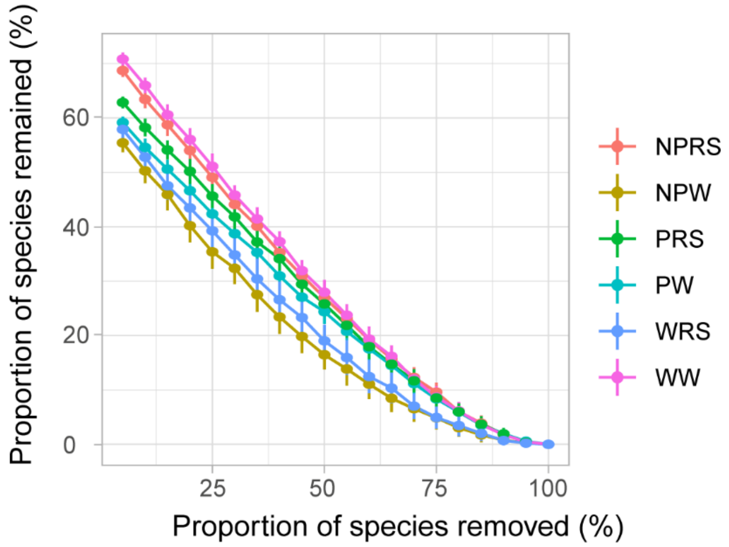

Supplementary Figure 4: The absolute abundance of 17 genera positively correlated with *R. solanacearum* varied across samples. Absolute abundances were converted by multiplying the 16S rRNA gene qPCR quantification results by the relative abundance of microorganisms. Abscissa represent the 17 genera, and ordinate coordinates represent the absolute abundance. The dots represent the values of absolute microbial abundance in different samples, and the curves represent the results of fitting the changes in microbial abundance. NPW: non-prebiotic + water, NPRS: non-prebitic + *R. solanacearum*, WW: water + water, PW: prebiotics + water, PRS: prebiotic + *R. solanacearum*, WRS: water + *R. solanacearum*.

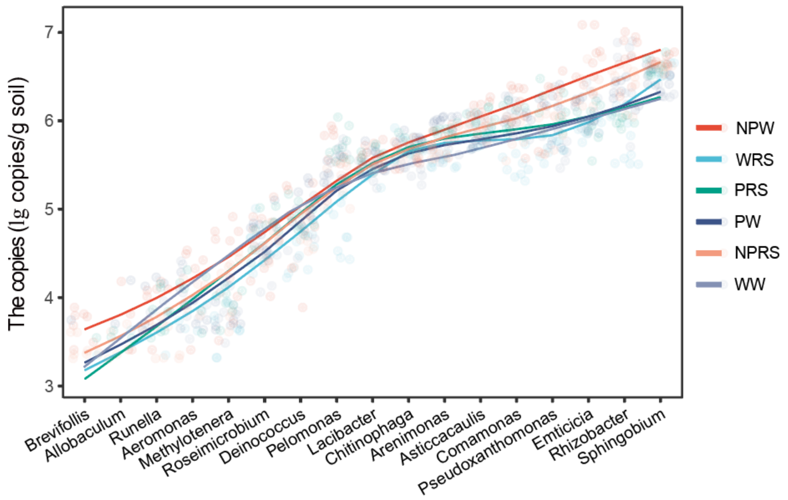

Supplementary Figure 5 Boxplots show the incidence of bacterial wilt in sterile tomato seedling. NPRS: non-prebitic + *R. solanacearum*, PRS: prebitic + *R. solanacearum*, WRS: water + *R. solanacearum*. Horizontal bars within boxes represent the median. The tops and bottoms of boxes represent 75th and 25th quartiles, respectively. The upper and lower whiskers represent the range of non-outlier data values. Outliers were plotted as individual points. Different lowercase letters indicated significant differences among respective groups based on two-sided tests for multiple comparisons by Turkey HSD corrections (t-test, adjusted $p < 0.05$, n = 8 biologically independent samples).

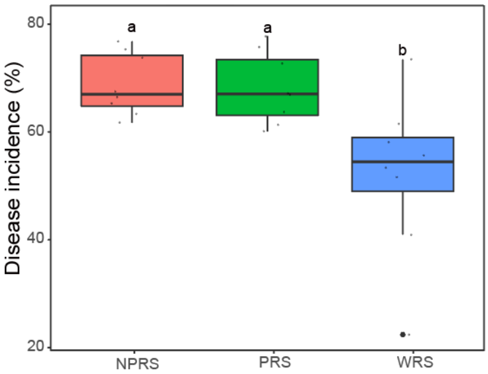

Supplementary Figure 6 The copy numbers of *fliC* and 16S rRNA gene in field tomato rhizosphere. Horizontal bars within boxes represent the median. The tops and bottoms of boxes represent 75th and 25th quartiles, respectively. The upper and lower whiskers represent the range of non-outlier data values. Outliers were plotted as individual points. Different lowercase letters indicated significant differences among respective groups based on two-sided tests for multiple comparisons by Turkey HSD corrections (t-test, adjusted $p < 0.05$, n = 8 biologically independent samples).

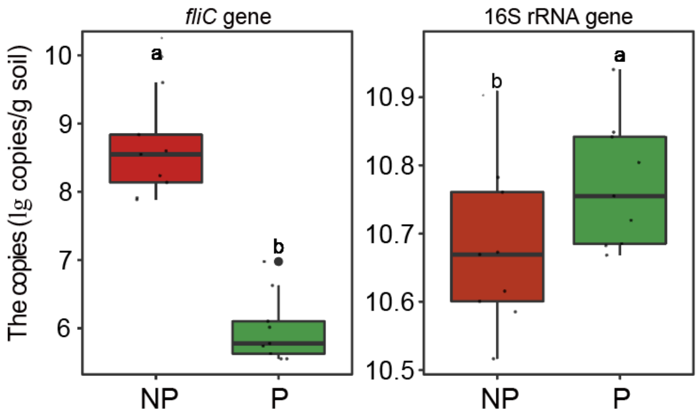

Supplementary Figure 7 *Alpha* diversity of microorganisms involved in the functional enrichment of prebiotics. Horizontal bars within boxes represent the median. The tops and bottoms of boxes represent 75th and 25th quartiles, respectively. The upper and lower whiskers represent the range of non-outlier data values. Outliers were plotted as individual points. Different lowercase letters indicated significant differences among respective groups based on two-sided tests by Wilcoxon rank-sum test followed by Dunn's multiple comparison test (adjusted $p < 0.05$, n = 6 biologically independent samples).

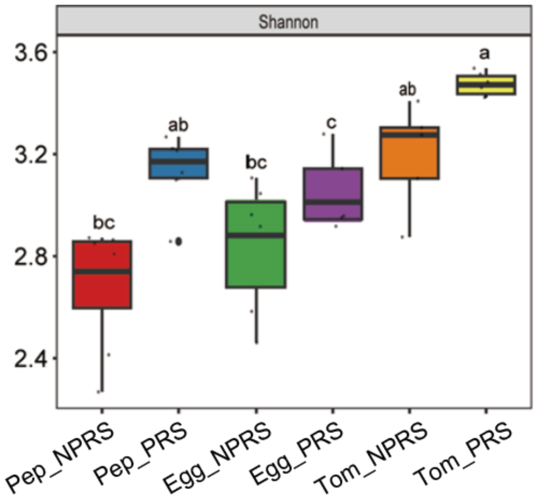

Supplementary Table 1 The relative abundance of the identified metabolite groups between healthy and diseased samples.

	Healthy1	Healthy2	Healthy3	Healthy4	Diseased1	Diseased2	Diseased3	Diseased4
Alcohols	0.1234	0.1357	0.1262	0.0497	0.2267	0.2446	0.1698	0.1400
Amino acids and amides	0.0053	0.0074	0.0048	0.0048	0.0073	0.0073	0.0079	0.0071
Esters	0.0680	0.0935	0.0810	0.1026	0.1125	0.1113	0.1243	0.1257
Long chain carbon organic acids	0.2881	0.2070	0.2286	0.1959	0.5161	0.4991	0.5439	0.5182
Nucleotides	0.0011	0.0012	0.0017	0.0069	0.0008	0.0008	0.0010	0.0041
Others	0.1349	0.1367	0.0915	0.1271	0.0566	0.0602	0.0682	0.1116
Short chain carbon organic acids	0.0392	0.0578	0.0567	0.0973	0.0177	0.0176	0.0205	0.0212
Sugar acids	0.0007	0.0006	0.0007	0.0011	0.0017	0.0018	0.0020	0.0013
Sugar alcohols	0.0367	0.0381	0.0269	0.0287	0.0214	0.0072	0.0185	0.0136
Sugars	0.3026	0.3220	0.3819	0.3859	0.0391	0.0501	0.0439	0.0573

Supplementary Table 2 The result of LMs analysis in tomato disease. The statistical tests were conducted at two-sided 5% significance level.

Day	DFn	DFd	F	P value	R squared
6	2	21	22.27	6.47E-06	0.68
7	2	21	11.86	0.000358	0.53
8	2	21	25.58	2.35E-06	0.71
9	2	21	26.03	2.07E-06	0.71
10	2	21	44.38	2.88E-08	0.81
11	2	21	45.19	2.47E-08	0.81
12	2	21	44.15	3.01E-08	0.81
13	2	21	32.95	3.34E-07	0.76
14	2	21	28.22	1.12E-06	0.73
15	2	21	29.10	8.85E-07	0.74
16	2	21	82.05	1.19E-10	0.89

Supplementary Table 3 LMs analysis results of the copy numbers of *fli*C and 16S rRNA genes. The statistical tests were conducted at two-sided 5% significance level.

Group	DFn	DFd	F	P value	R squared
*fli*C	5	30	139.87	1.04E-12	0.96
16S rRNA gene	5	30	13.08	8.5E-07	0.69

Supplementary Table 4 The result of two-factors permutational multivariate analysis of variance (two-way PERMANOVA, $p < 0.05$). The *post*-hoc pairwise tests with Turkey HSD.

	Df	SumOfSqs	R^2	F	P value
Pathogen	1	0.17977	0.06994	3.7633	0.01
Prebiotics	1	0.72256	0.28112	15.1257	0.001
Pathogen & Prebiotics	1	0.13926	0.05418	2.9153	0.006
Residual	32	1.52865	0.59475		
Total	35	2.57025	1		

Supplementary Table 5 Two-way PERMANOVA (p < 0.05) *post*-hoc pairwise tests with Turkey HSD.

ID	Stat	P.adj
NPRS VS_NPW	ADONIS.r 0.974	0.002
NPRS VS PRS	ADONIS.r 0.91	0.001
NPRS VS PW	ADONIS.r 0.933	0.001
NPRS VS WRS	ADONIS.r 0.641	0.001
NPRS VS WW	ADONIS.r 0.457	0.003
NPW VS PRS	ADONIS.r 0.825	0.001
NPW VS PW	ADONIS.r 0.712	0.001
NPW VS WRS	ADONIS.r 0.633	0.004
NPW VS WW	ADONIS.r 0.822	0.001
PRS VS PW	ADONIS.r 0.313	0.002
PRS VS WRS	ADONIS.r 0.711	0.001
PRS VS WW	ADONIS.r 0.722	0.003
PW VS WRS	ADONIS.r 0.707	0.001
PW VS WW	ADONIS.r 0.666	0.002
WRS VS WW	ADONIS.r 0.683	0.001

Supplementary Table 6 Global network properties of co-occurrence network analyses in different treatments.

	NPW	NPP	WW	PW	PRS	WRS
Num.edges (L)	147.00	1158.00	9175.00	597.00	493.00	143.00
Num.pos.edges	135.00	949.00	8809.00	532.00	452.00	118.00
Num.neg.edges	12.00	209.00	366.00	65.00	41.00	25.00
Num.vertices (n)	132.00	307.00	452.00	248.00	262.00	113.00
Connectance	0.02	0.02	0.09	0.02	0.01	0.02
Average.degree	2.23	7.54	40.60	4.81	3.76	2.53
Average.path.length	3.34	4.95	3.69	4.37	8.22	3.97
Diameter	9.58	14.84	11.43	13.15	18.86	10.55
Edge.connectivity	0.00	0.00	0.00	0.00	0.00	0.00
Mean.clustering.coefficient	0.47	0.48	0.56	0.42	0.40	0.56
No.clusters	35.00	31.00	5.00	31.00	38.00	24.00
Centralization.degree	0.07	0.13	0.22	0.08	0.07	0.05
Centralization.betweenness	0.04	0.08	0.05	0.08	0.09	0.05
Centralization.closeness	0.00	0.00	0.02	0.00	0.00	0.00
Rm	0.33	0.29	0.43	0.62	0.59	0.51

Supplementary Table 7 A total of 474 commensal microbes, which negatively correlated (35) or not related (439) with *R. solanacearum*, were picked with the spearman method with the BH correction. Also, the number of positively microbes was 17.

Correlation with *R. solanacearum*	Number	P value	Post-hoc
Positive	17	< 0.05	BH
Negative	35	< 0.05	BH
None	439	> 0.05	BH

Supplementary Table 8 LMs analysis results of the dilution plating of *R. solanacearum* and SynCom among different rhizosphere samples. The statistical tests were conducted at two-sided 5% significance level.

Group	Day	DFn	DFd	F	P value	R squared
R. solanacearum	1	4	29	16.00	5.05E-07	0.69
R. solanacearum	3	4	30	9.78	3.48E-05	0.57
R. solanacearum	5	4	29	26.41	2.66E-09	0.79
SynCom	1	4	29	24.54	6.00E-09	0.77
SynCom	3	4	30	25.63	2.65E-09	0.77
SynCom	5	4	29	6.62	0.00065	0.48

Supplementary Table 9 LMs analysis results of the disease incidence of three *Solanaceae* crops. The statistical tests were conducted at two-sided 5% significance level.

Day	DFn	DFd	F	P value	R squared
10	2	69	15.89	5.05E-07	0.32
15	2	69	36.01	3.48E-05	0.51
20	2	69	88.02	2.66E-09	0.72
25	2	69	105.46	6.00E-09	0.75
30	2	69	122.63	2.65E-09	0.78

Supplementary Table 10 LMs analysis results of the copies number 16S rRNA and *fli*C gene among three *Solanaceae* crops. The statistical tests were conducted at two-sided 5% significance level.

Plant	qPCR	DFn	DFd	F	P value	R squared
Pepper	16S rRNA gene	1	14	9.135	0.009	0.40
Eggplant	16S rRNA gene	1	14	4.404	0.036	0.27
Tomato	16S rRNA gene	1	14	4.235	0.032	0.21
Pepper	*fli*C	1	14	6.471	0.024	0.30
Eggplant	*fli*C	1	14	6.662	0.022	0.32
Tomato	*fli*C	1	14	73.787	5.93E-07	0.84
