# Supplementary material for: Tapping the rhizosphere metabolites for the prebiotic control of soil-borne bacterial wilt disease
Source: Nat Commun. 2023 Jul 26;14:4497. doi: 10.1038/s41467-023-40184-2 (PMC10372070; doi:10.1038/s41467-023-40184-2)
Supplement: Supplementary file 3 — Description of Additional Supplementary Files [file 41467_2023_40184_MOESM3_ESM.pdf]

File Name: Supplementary Data 1

Description: Metabolites that have different abundances in the healthy and diseased tomato rhizosphere. The statistical tests were conducted at two-sided 5% significance level with Wald applied for multiple comparisons (n = 4 biologically independent samples).
